# Different Oxidative Stress Response in Keratinocytes and Fibroblasts of Reconstructed Skin Exposed to Non Extreme Daily-Ultraviolet Radiation

**DOI:** 10.1371/journal.pone.0012059

**Published:** 2010-08-10

**Authors:** Claire Marionnet, Cécile Pierrard, François Lejeune, Juliette Sok, Marie Thomas, Françoise Bernerd

**Affiliations:** L'Oréal Recherche, Clichy, France; Buck Institute for Age Research, United States of America

## Abstract

Experiments characterizing the biological effects of sun exposure have usually involved solar simulators. However, they addressed the worst case scenario i.e. zenithal sun, rarely found in common outdoor activities. A non-extreme ultraviolet radiation (UV) spectrum referred as “daily UV radiation” (DUVR) with a higher UVA (320–400 nm) to UVB (280–320 nm) irradiance ratio has therefore been defined. In this study, the biological impact of an acute exposure to low physiological doses of DUVR (corresponding to 10 and 20% of the dose received per day in Paris mid-April) on a 3 dimensional reconstructed skin model, was analysed. In such conditions, epidermal and dermal morphological alterations could only be detected after the highest dose of DUVR. We then focused on oxidative stress response induced by DUVR, by analyzing the modulation of mRNA level of 24 markers in parallel in fibroblasts and keratinocytes. DUVR significantly modulated mRNA levels of these markers in both cell types. A cell type differential response was noticed: it was faster in fibroblasts, with a majority of inductions and high levels of modulation in contrast to keratinocyte response. Our results thus revealed a higher sensitivity in response to oxidative stress of dermal fibroblasts although located deeper in the skin, giving new insights into the skin biological events occurring in everyday UV exposure.

## Introduction

Chronic sun exposure is responsible for long term clinical skin changes such as photoaging and photocancers [1], [2]. These effects have been mostly attributed to the deleterious impact of ultra-violet (UV) radiation involving a combination of UVB (280–320 nm) and UVA (320–400 nm) wavelengths. In order to experimentally assess the effects of solar UV, standard UV spectra have been defined [3]. However they represent extreme solar UV exposure conditions with a quasi zenithal sun irradiance i.e. a UVA to UVB irradiance ratio of less than 18, representative of a high UVB level. In these conditions even a short time exposure leads to an erythemal sunburn reaction reflecting the direct impact of UVB, i.e. DNA lesions, apoptotic sunburn keratinocytes, accumulation of P53 [4]. However, the solar UV spectrum reaching earth depends on many parameters including latitude, season, time of day, meteorological conditions or ozone layer thickness. Therefore zenithal sun exposure conditions, corresponding to summer sunlight at noon and maximizing UVB proportion are rarely found. In addition, suberythemal repetitive doses of solar UV have been shown to induce damage that might result in long term development of photoaging and photocancers [5], [6]. Several studies have also proven that UVA wavelengths by themselves participated in these long term clinical effects [7], [8]. To assess more realistic solar UV exposure, a non-zenithal UV spectrum has been defined as standard daily ultraviolet radiation (DUVR) spectrum, with a UVA to UVB irradiance ratio of around 27 [9]. Repetitive exposures to a low sub-erythemal DUVR dose for 19 consecutive days modified biological parameters in both the epidermis and the dermis of human skin [10]. Altogether these results emphasized the importance of spectral distribution of the UV spectrum with regards to biological effects in both skin compartments.

DUVR spectrum includes a high and constant proportion of UVA wavelengths, known to stimulate the production of reactive oxygen species (ROS) that play a major role in photoaging. For example ROS lead to an increased expression of matrix-metalloproteinases resulting in degradation of the dermal connective tissue [11] and induce common deletion mutation of mitochondrial DNA, a molecular hallmark of photoaging [12]. To protect itself from oxidative stress, the skin has developed several defense systems, including ROS and metal ions scavengers and a battery of detoxifying and repair enzymes [13]–[15]. In addition, UVA can also directly induce DNA strand breaks, which in turn can influence various intracellular signaling, including oxidative stress responsive genes [16]–[17].

The aim of the present study was to analyze the impact of oxidative stress induced by a single DUVR exposure in the reconstructed skin model composed of both a living dermal equivalent and a fully differentiated epidermis. This model provides a useful tool to study keratinocyte and fibroblast responses in a three dimensional context which is more physiological than typical skin cell culture. Two physiological doses were chosen, 7 and 13 J/cm^2^ DUVR, corresponding respectively to 10 and 20% of the dose received per day in Paris on mid-April [10]. After the study of the impact of DUVR on the morphology of human reconstructed skin, the gene expression of 24 markers involved in antioxidant cell response was assessed in parallel in fibroblasts and keratinocytes of the reconstructed human skin by quantitative reverse transcription-polymerase chain reaction (RT-PCR) after DUVR exposure. Studied markers included superoxide dismutase (SOD1 and SOD2), catalase, thioredoxin (TXN), metallothioneins (MT1X, MT1G, MT1E, MT2A), actors (NF-E2-related factor 2 (Nrf2), kelch-like ECH-associated protein 1 (Keap1), BTB and CNC homology 1, basic leucine zipper transcription factor 1 (Bach 1)) and targets (glutathione peroxidase 1 (GSH Px), thioredoxine reductase (TXNR), NADPH:quinone oxidoreductase (NQO1), heme oxygenase-1 (HO-1), glutamylcysteine synthetase light and heavy chains (γ GCS- L, γ GCS- H), ferritins heavy and light chains (FTH and FTL)) of the (Nrf2) pathway, sestrins (SESN1-T1, SESN1-T2, SESN2, SESN3) and methionine sulfoxide reductase A (MSRA).

## Results

### DUVR source

**Figure 1:**
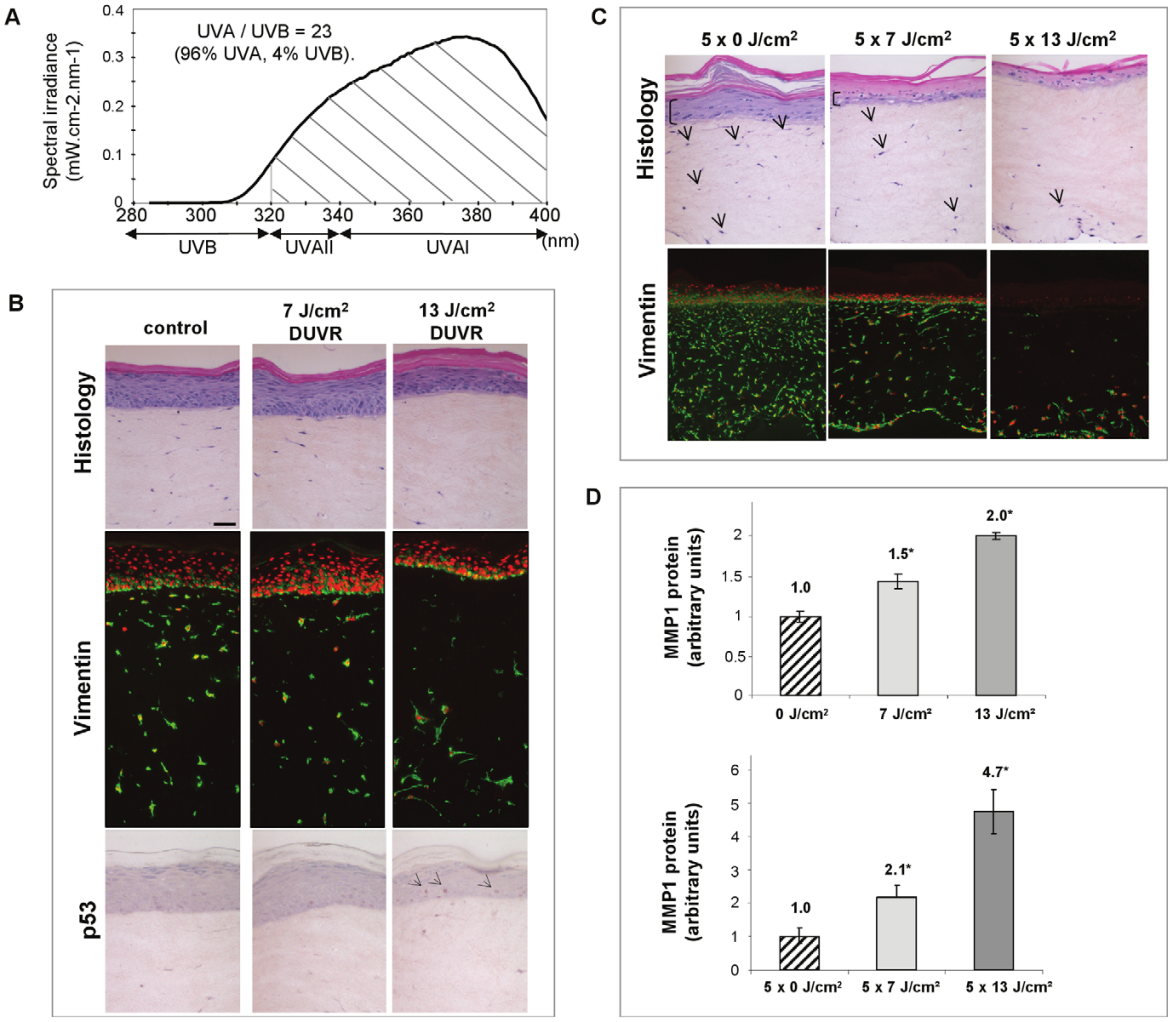
Tissular and cellular effects of simulated UV daylight on human reconstructed skin. (a): DUVR spectrum was recorded with a calibrated Macam spectroradiometer. Hatched area represents UVA portion in DUVR spectrum. (b): Sham irradiated (control), 7 J/cm^2^ and 13 J/cm^2^ DUVR exposed samples were taken for classical histology and for vimentin (vimentin: green labelling, nuclei counterstaining: red labelling) and p53 immunolabelling at 48 h. Exposure to 7 J/cm^2^ DUVR did not induce changes compared to the control sample. In contrast, 13 J/cm^2^ DUVR led to the disappearance of superficial dermal fibroblasts and to the induction of p53 positive keratinocytes (arrows). (c) Samples were sham irradiated (control) or exposed to 7 J/cm^2^ and 13 J/cm^2^ DUVR once a day for 5 consecutive days. They were sampled for histology and vimnetin immunostaining 72 h after the last exposure. Compared to controls, samples treated with repeated doses of DUVR showed drastic alterations in epidermis (decreased thickness and death of suprabasal keratinocytes) and dermis (decrease in the number of fibroblasts). Arrows indicate living fibroblasts, brackets show living keratinocytes. (d): MMP-1 ELISA assay : the amount of MMP-1 produced in the culture medium was measured after acute or repeated exposures (once a day for 5 consecutive days) to DUVR at the doses indicated. Asteriks indicate significant different values. Bar = 50 µm.

To mimic standard DUVR, which represents non-extreme exposure conditions, the simulated DUVR spectrum was used (Figure 1a). The UVA (320–400 nm) to UVB (290–320 nm) irradiance ratio was 23. It was adequate to consider the irradiation spectrum as a good simulation of standard DUVR [9] and represented a UV distribution of 4% in UVB and 96% in UVA.

### Damage in reconstructed skin morphology after DUVR exposure

Reconstructed human skin was exposed to DUVR (7 J/cm^2^ or 13 J/cm^2^). 13 J/cm^2^ DUVR had an impact on both compartments of reconstructed skin with the disappearance of dermal superficial fibroblasts, slight morphological alterations in the epidermis such as thining of the epidermis and thickening of the cornified layer, and the appearance of p53 positive keratinocytes. For the lower dose (7 J/cm^2^ DUVR), the global architecture of both skin compartments was not altered and no P53 accumulation was detected (Figure 1b).

In order to determine the effect of repeated exposure to DUVR, as a of “chronic” exposure model, reconstructed skins were exposed to 7 or 13 J/cm^2^ DUVR every day for 5 consecutive days. For both doses, drastic alterations could be observed in the epidermis and in the dermis suggesting that these exposures may be responsible for long term alterations (Figure 1c).

Matrix Metalloproteinase 1 (MMP-1), another well known marker for photoaging also showed an increase in its production after acute or repetitive exposures to both of the DUVR doses tested (Figure 1d).

### Exposure of reconstructed skin to DUVR led to oxidative stress in epidermal keratinocytes and dermal fibroblasts

**Figure 2:**
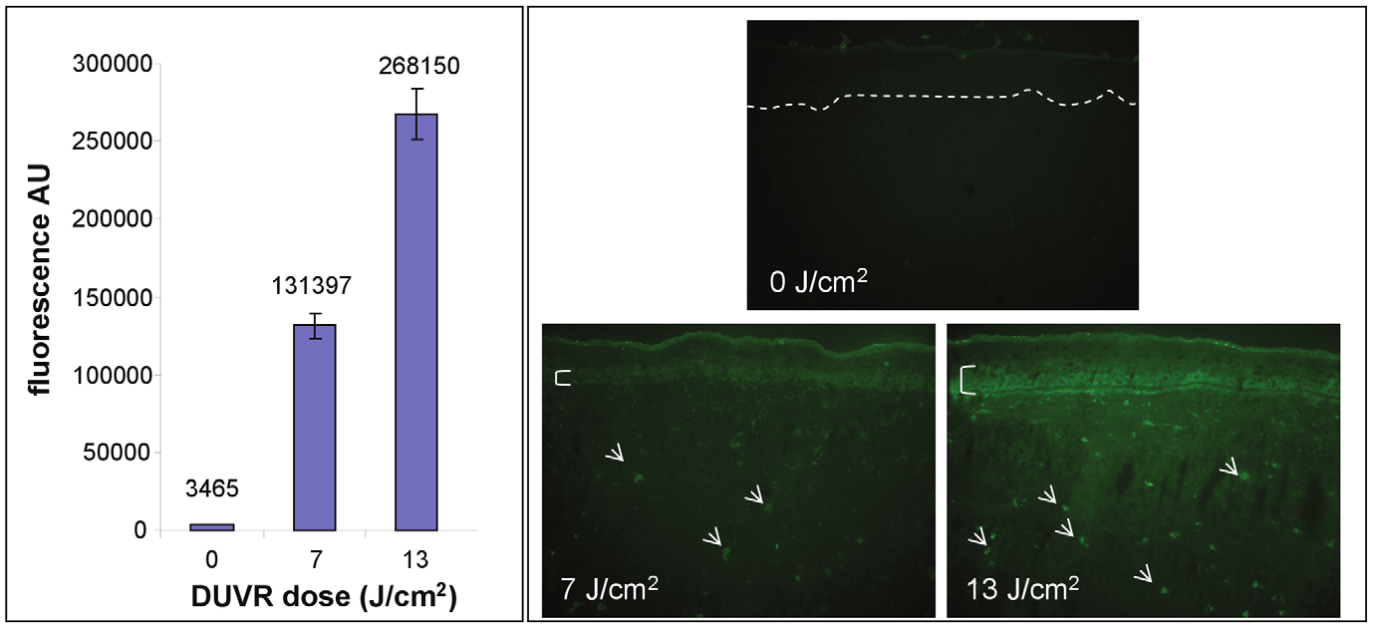
ROS assay in reconstructed skin exposed to DUVR. Left pannel - Levels of DCFH-DA fluorescence in reconstructed skin after DUVR exposure. Right pannel - Sections of reconstructed skin after DCFH-DA probe incorporation and DUVR exposure. Bracket and arrows indicated the fluorescent keratinocytes and fibroblasts respectively in DUVR-exposed samples. None of them were detected in non-exposed reconstructed skin. Dotted line delimitates epidermis and dermis.

The generation of ROS induced by such DUVR exposure of reconstructed skin was quantified and visualized after incorporation of a 2′,7′-dichlorodihydrofluorescein diacetate (DCFH-DA) probe (figure 2). DUVR exposure led to a dose dependent increase in ROS production in reconstructed skins (38-fold and 77-fold increase at 7 J/cm^2^ and 13 J/cm^2^ respectively compared to non exposed control). Both fibroblasts and keratinocytes did produce ROS after DUVR exposure as seen on skin cryosections (figure 2).

DUVR also led to a significant increase in epidermal concentration of lipid peroxides (data not shown).

### Exposure of reconstructed skin to DUVR led to a modulation in the amount of mRNA of markers involved in oxidative stress and revealed a differential response between fibroblasts and keratinocytes

To assess oxidative stress response at the gene expression level, mRNA of 24 markers were quantified in fibroblasts and keratinocytes isolated from the reconstructed skin, at three time points following a DUVR exposure. Modulation ratios (mRNA amount of irradiated sample to mean mRNA amount of the 3 control samples) were calculated for each marker and at each time point in both cell types.

**Figure 3:**
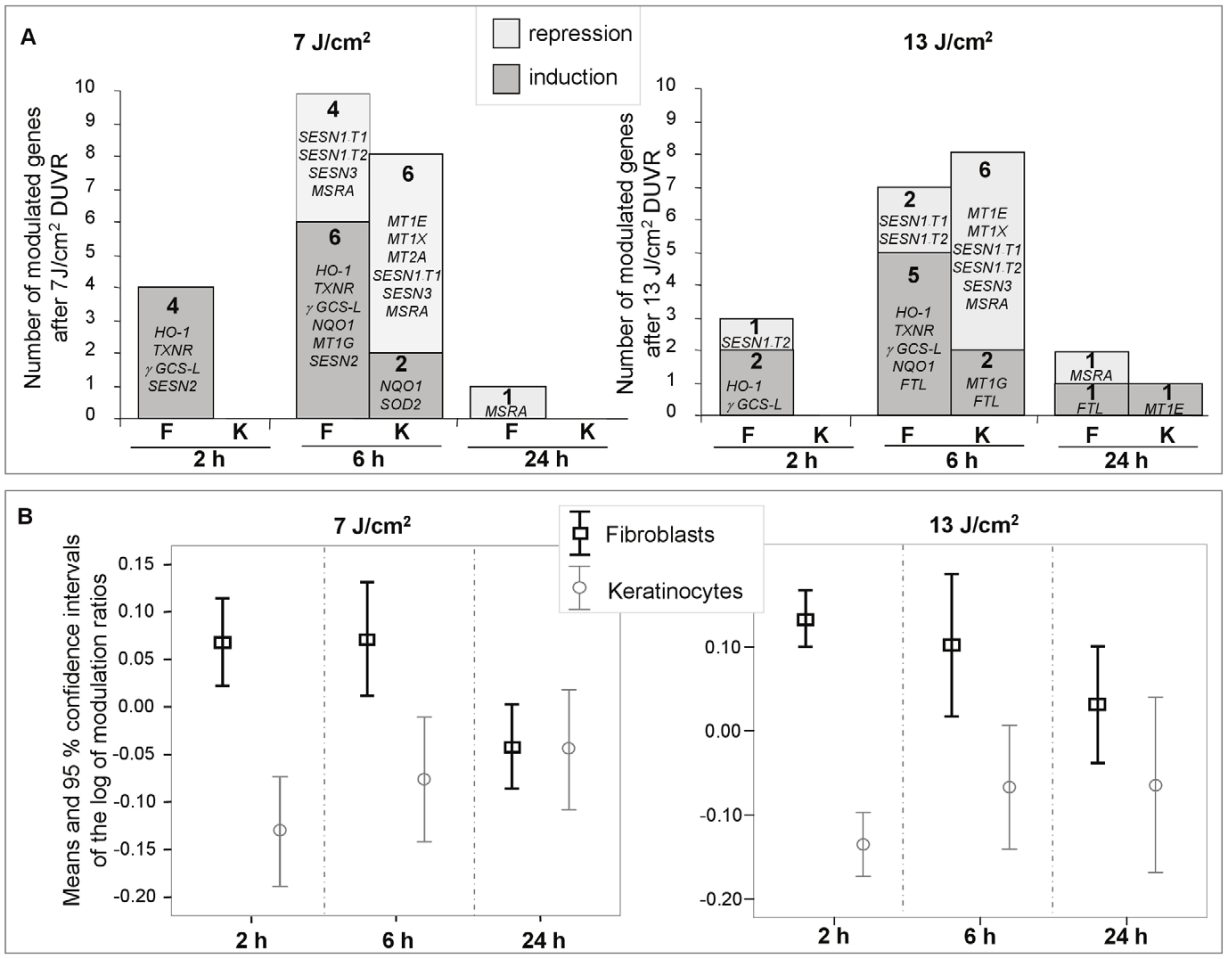
Distribution, type and mean of gene modulation after DUVR exposure of human reconstructed skin. 2, 6 and 24 hours after DUVR exposure, mRNA levels of 24 oxidative stress markers were quantified by QPCR in fibroblasts (F) and keratinocytes (K) of reconstructed skin separately, in three independent experiments per time point. (a) Number and type of modulation of significantly modulated genes. (b) Means and confidence intervals of the log of modulation ratios. At each time point, gene modulation ratios (mRNA amount in exposed sample to mean mRNA amount in the 3 control samples) were calculated for each marker. Means and confidence intervals of the 24 log_10_ of modulation ratio were calculated. Values higher or lower than 0, correspond to induction or repression of mRNA amount, respectively.

In reconstructed skin, 7 and 13 J/cm^2^ of DUVR led to significant gene expression modulation of oxidative stress markers in both fibroblasts and keratinocytes (p<0.05). Compared to keratinocytes, fibroblasts exhibited a higher number of significant gene modulations after DUVR exposure (15 *vs* 8 after 7 J/cm^2^ and 12 *vs* 9 after 13 J/cm^2^ DUVR) (Figure 3a). Moreover, regarding the type of gene modulation, DUVR mostly led to inductions in fibroblasts (67% of modulations) and to repressions in keratinocytes (reaching 75% of modulations after 7 J/cm^2^ DUVR). The maximum number of significant gene modulations was found 6 hours after DUVR exposure (7 or 13 J/cm^2^) in keratinocytes and in fibroblasts (Figure 3a). In keratinocytes, no modulation was found at 2 hours while fibroblasts exhibited earlier gene modulation, with 4 and 3 gene modulations 2 hours after respectively 7 and 13 J/cm^2^ DUVR (Figure 3a).

Analysis of the overall effect of DUVR exposure by a two-way ANOVA test on the set of the 24 tested genes showed that fibroblast response was significantly different from that of keratinocytes (p<0.0001). The ANOVA test was then applied at each time point and revealed that at 2 and at 6 hours following DUVR exposure, significant higher values of modulation ratios were found in fibroblasts compared to keratinocytes (at 2 hours, p<0.0001 for any dose of DUVR; at 6 hours p = 0.004 for 7 J/cm^2^ and p = 0.08 for 13 J/cm^2^). No significant difference was found at 24 hours (Figure 3b).

### DUVR induced modulation of different families of oxidative stress markers in reconstructed skin

A detailed analysis of different gene families involved in oxidative stress management was then performed. At any tested dose, DUVR did not alter mRNA level of catalase, thioredoxin, glutathion peroxidase, or the transcription factor Nrf2 and its modulators Keap1 and Bach1. Altered gene expression was mostly found after DUVR exposure in 4 families of genes, namely Nrf2 target genes, sestrins, metallothioneins and MSRA.

#### Nrf2 target genes: HO-1, TXNR, NQO-1, γ GCS-L and γ GCS-H

**Figure 4:**
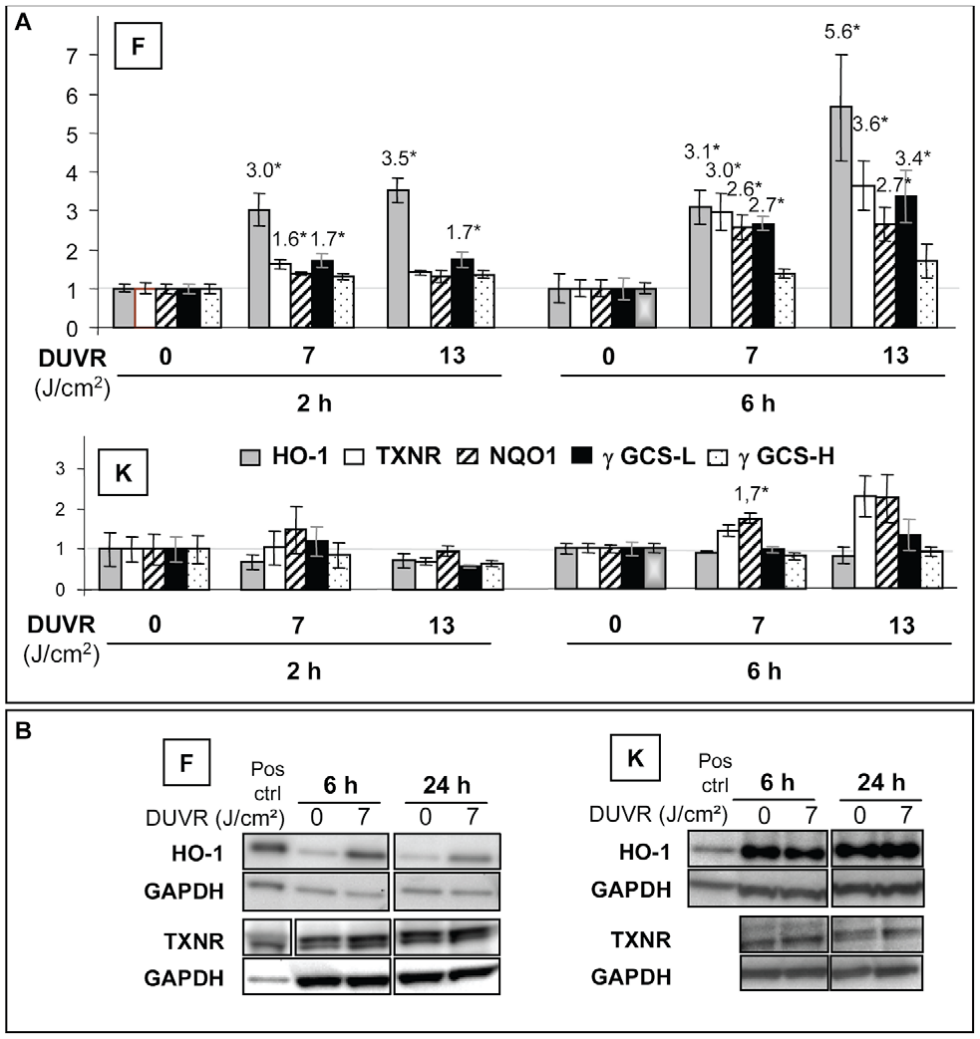
Modulation of mRNA and protein levels of Nrf2 target genes in reconstructed skin exposed to DUVR. (a): 2 and 6 hours after DUVR exposure (7 or 13 J/cm^2^), mRNA levels of HO-1, TXNR, NQO1, γ GCS-L and γ GCS-H were quantified by QPCR in fibroblasts (F) and keratinocytes (K). Each histogram bar shows the mean value ± standard error of mean (SEM) of normalized mRNA amount (n = 3). mRNA amount in sham-exposed samples was adjusted to the 1 value. Indicated values correspond to significant modulations (*, p<0.05). (b): 6 and 24 hours after 7 J/cm^2^ DUVR, HO-1 and TXNR protein levels, respectively present in whole cell and cytosolic extracts, were determined by western blot in fibroblasts (F) and keratinocytes (K) of reconstructed skin. GAPDH levels were used to normalize data. Positive control: normal human melanocytes treated with 20 µM forskolin.

In fibroblasts of reconstructed skin, DUVR led to significant up-regulation of four out of the five tested Nrf2 target genes. As early as 2 hours post exposure, HO-1, TXNR and γ -GCS-L mRNA were induced by 7 J/cm^2^ DUVR exposure compared to control samples with induction levels of 3, 1.6 and 1.7 respectively. This fibroblast response was amplified 6 hours post exposure, in terms of level of modulation and with the supplementary induction of NQO1. At 13 J/cm^2^, the same genes were induced with higher induction levels (Figure 4a). At 24 hours, all mRNA levels of these genes returned back to basal level (data not shown).

In epidermal keratinocytes, only NQO1 mRNA level was significantly induced (×1.7) 6 hours after exposure to 7 J/cm^2^ DUVR.

At time points 2 and 6 hours, means of modulation ratios of Nrf2 target genes were significantly different between fibroblasts and keratinocytes for 7 and 13 J/cm^2^ DUVR (ANOVA, p<0.001, for any time and for any dose).

Protein levels of two markers, i.e. HO-1 and TXNR showed a good correlation with mRNA data (Figure 4b). HO-1 protein levels were strongly induced 6 hours after exposure to 7 J/cm^2^ DUVR in fibroblasts of reconstructed skin. This induction was sustained at 24 hours. No modulation of HO-1 protein could be detected in keratinocytes of reconstructed skin exposed to DUVR.

TXNR protein levels were slightly induced in fibroblasts at 6 and 24 hours after 7 J/cm^2^ DUVR (×1.3) whereas no modulation could be detected in keratinocytes.

#### Sestrins: SESN1-T1, SESN1-T2, SESN2, SESN3

**Figure 5:**
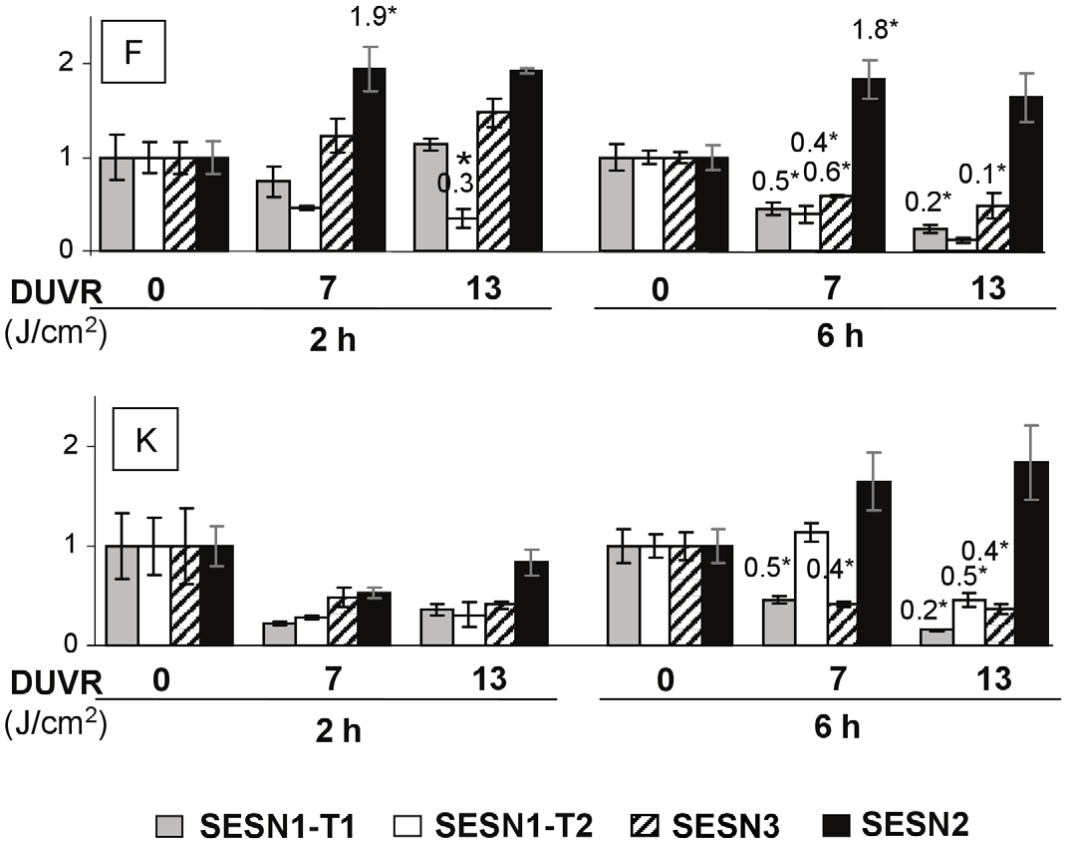
Effects of DUVR on mRNA levels of sestrins in human reconstructed skin. At 2 hours and 6 hours after DUVR exposure, mRNA levels of sestrins genes i.e. SESN1-T1, SESN1-T2, SESN3, SESN2 were quantified by QPCR in fibroblasts (F) and keratinocytes (K) of reconstructed skin, in sham or DUVR exposed samples (7 or 13 J/cm^2^). Each histogram bar shows the mean value ± SEM of normalized mRNA amount (n = 3). mRNA amount of each marker in sham-exposed samples was adjusted to the 1 value. Indicated values correspond to means of mRNA amount in DUVR exposed samples statistically different from means of mRNA amount in control samples (*, Student't test, p<0.05).

Sestrin family includes 3 genes: SESN1 encoding 3 transcripts (SESN1-T1, -T2 and T3) [18], SESN2 [19], [20] and SESN3, encoding a hypothetical protein [21]. In fibroblasts, SESN2 was induced by 2-fold 2 and 6 hours after 7 J/cm^2^ DUVR exposure. This induction was also observed 2 hours after 13 J/cm^2^ although it was not significant (p = 0.058). In contrast, mRNA levels of the other sestrins tested (SESN1-T1, SESN1-T2 and SESN3) were significantly down-regulated at 6 hours post DUVR in fibroblasts and keratinocytes (Figure 5). In both cell types, levels of sestrins mRNA were no longer modulated at 24 h (data not shown).

#### Metallothioneins: MT1G, MT1X, MT1E, MT2

Four isoform families of Metallothioneins have been described. In human MT1 and MT2 isoforms are inducible. Seven functional MT1 genes are known and include MT1-E, MT1-G and MT1-X.

**Figure 6:**
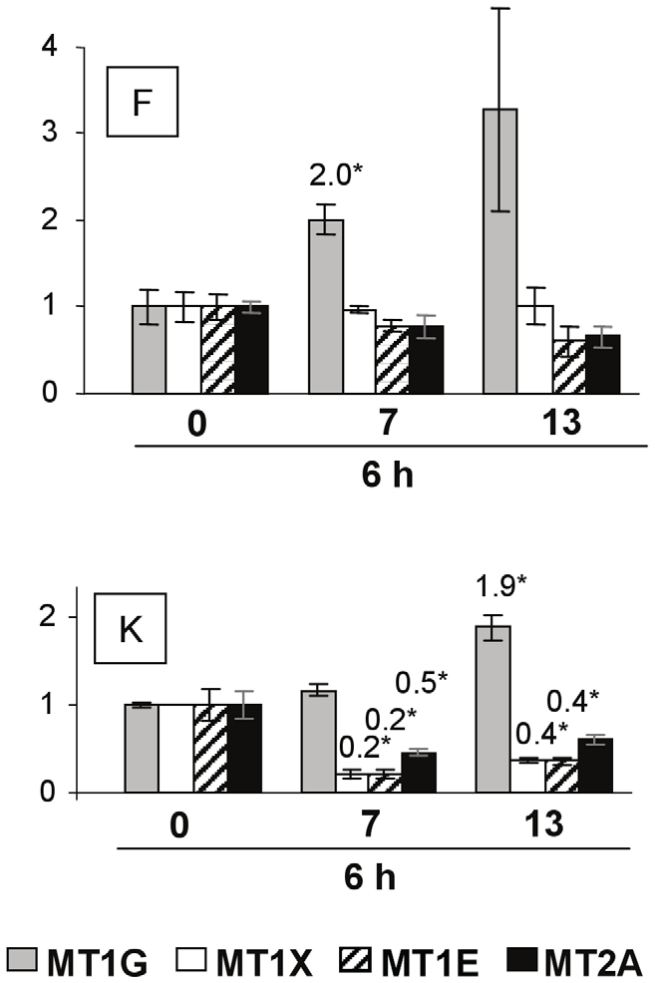
Effects of DUVR on mRNA levels of metallothionein subunits in human reconstructed skin. At 6 hours after DUVR exposure, mRNA levels of metallothionein genes i.e. MT1G, MT1X, MT1E and MT2A were quantified by QPCR in fibroblasts (F) and keratinocytes (K) of reconstructed skin, in sham or DUVR exposed samples (7 or 13 J/cm^2^). Each histogram bar shows the mean value ± SEM of normalized mRNA amount (n = 3). mRNA amount of each marker in sham-exposed samples was adjusted to the 1 value. Indicated values correspond to means of mRNA amount in DUVR exposed samples statistically different from means of mRNA amount in control samples (*, Student't test, p<0.05).

Metallothionein genes were modulated in fibroblasts and in keratinocytes only at the time point of 6 hours after DUVR exposure. MT1G was significantly induced in both cell types. Keratinocytes response was also characterized by a strong and significant down-regulation of MT1X, MT1E and MT2A mRNA for both DUVR doses (Figure 6). This repression was not found in fibroblasts, leading to different global modulation profiles of metallothioneins between both cell types, especially after 7 J/cm^2^ (ANOVA, p<0.0001).

### Methionine sulfoxide reductase: MSRA

**Figure 7:**
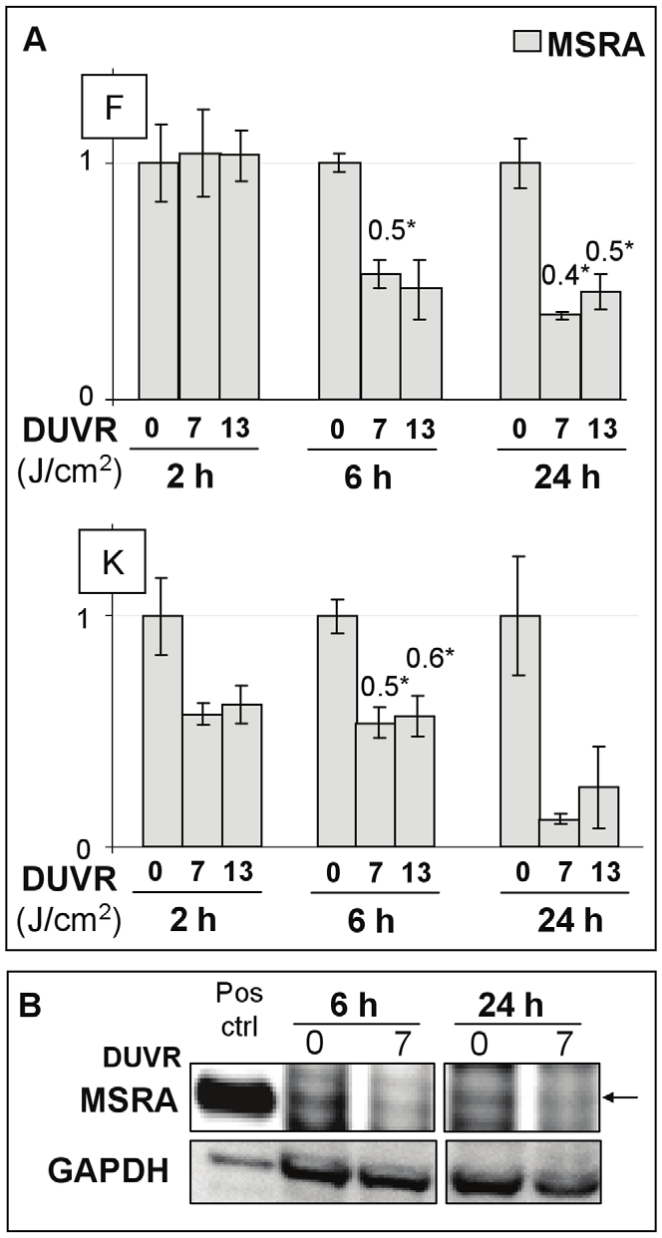
Effects of DUVR on MSRA mRNA and protein levels in human reconstructed skin. (a) 2, 6 and 24 hours after DUVR exposure (7 or 13 J/cm^2^), mRNA levels of MSRA were quantified by QPCR in fibroblasts (F) and keratinocytes (K) of reconstructed skin. Each histogram bar shows the mean value ± SEM of normalized mRNA amount (n = 3). mRNA amount of sham-exposed samples was adjusted to the 1 value. Indicated values correspond to significant modulations (see Material and Methods, * Student't test, p<0.05). (b) 6 and 24 hours after 7 J/cm^2^ DUVR, levels of MSRA protein was determined in whole cell extracts of epidermal keratinocytes of reconstructed skin. Levels of GAPDH were used to normalize data. The arrow indicates the band corresponding to MSRA.

In fibroblasts and keratinocytes of reconstructed skin, DUVR significantly reduced MSRA mRNA amount 6 hours after exposure. This repression was maintained at 24 hours post exposure and was still significant in fibroblasts (p<0.05) and, to a lower extent, in keratinocytes (p<0.1) (Figure 7a). Repression of MSRA at protein level was demonstrated in keratinocytes of reconstructed skin at 6 hours and 24 hours after 7 J/cm^2^ DUVR (Figure 7b). MSRA protein could not be detected in fibroblasts of reconstructed skin, in agreement with previous studies [22], [23].

## Discussion

The present study reports the effects of low UV exposure in a reconstructed skin model *in vitro*. UV spectrum and selected doses (7 and 13 J/cm^2^) were physiological and accounted for about 10% and 20% of the daily dose of UV received in Paris on mid-April (from 6 am to 8 pm: 68 J/cm^2^) and therefore could be considered as realistic received daily UV doses [24].

The reconstructed human skin *in vitro* has previously been shown to be a useful system to reproduce biological effects occurring after UV exposure, especially the sunburn related markers, or dermal damage associated with photoaging process [25]. Using these biological end-points, we showed that 7 J/cm^2^ DUVR was not responsible for their induction and found that 13 J/cm^2^ DUVR was required to do so, in full agreement with human *in vitro* and *in vivo* data (0.5 Minimum Erythemal Dose (MED)  = 7.6±1.4 J/cm^2^ DUVR) [10]; [26]. In addition, repetitive exposures clearly demonstrated that these low DUVR doses may account for long term deleterious consequences.

Our results illustrated that even after the very low dose of 7 J/cm^2^ DUVR (inducing no morphological skin alteration) a strong induction of ROS and a significant oxidative stress response was revealed in both epidermal and dermal tissues. This oxidative stress may be related to the high percentage of UVA wavelengths contained in the DUVR spectrum, which are well known inducers of ROS.

Our results revealed that fibroblast and keratinocyte responses to DUVR were different with regard to kinetics, direction or levels of modulation, and nature of modulated genes. In dermal fibroblasts, oxidative stress response occurred rapidly, as early as 2 hours post exposure, with a majority of inductions. In contrast, modulated genes in keratinocytes were mostly found at 6 hours post exposure with a higher percentage of down-regulations.

The analysis of modulated genes revealed that numerous Nrf2 target genes, including HO1, TXNR, NQO1 and γ GCS-L were up-regulated in fibroblasts while only NQO1 was slightly (but significantly) induced in keratinocytes. The absence of HO-1 gene and protein induction in normal human keratinocytes is in line with previous studies [27]. Regarding the other Nrf2 target genes, very few studies have been performed in normal human keratinocytes and contradictory results may be found in immortalized keratinocytes [28], [29]. Here we show that in an organotypic 3D model, normal human epidermal keratinocytes are able to induce at least one Nrf2 target gene after exposure to a low dose of DUVR, but to a lower extent than dermal fibroblasts. Other gene families such as metallothioneins (MT) were differentially modulated in fibroblasts and keratinocytes. MT are small proteins with a high cysteine content and have an important role in scavenging ROS and metal ions [15]. MT-1 and MT-2 are rapidly and highly inducible by a variety of stimuli including metals, hormones, cytokines, oxidants, stress and UV [30]. In agreement with these results, we showed that MT1G gene expression was induced in human dermal fibroblasts of reconstructed skin exposed to DUVR. However, keratinocytes behaved differently under the same exposure, with repression of MT1X, MT1E and MT2A mRNA levels. In line with these results, a bi-phasic response of keratinocytes to UVC/UVB wavelengths, with a rapid down-regulation of MT gene transcription and a subsequent enhancement, has been reported [31], [32]. The down-regulation of MT may result in a transient increase in UV sensitivity because of the photoprotective role of MT highly suggested by data obtained in MT null mice [33], [34]. Finally, SESN2 gene expression was also differentially regulated after DUVR in both cell types with induction in dermal fibroblasts and no effect in keratinocytes. It was previously shown that SESN2 was inducible by oxidative or genotoxic stress in several human cell lines [18], [19].

Altogether these results gave clear evidence of distinct responses to oxidative stress between fibroblasts and keratinocytes which could reflect differences in basal anti-oxidant defense equipment [27], [35]–[38]. Previous data obtained in reconstructed skin model also revealed that survival ability of dermal fibroblasts was lower compared to epidermal keratinocytes after exposure to pure UVA. This effect has been shown to be related to an apoptotic process only occuring in dermal fibroblasts compared to epidermal keratinocytes [25]. Variations in responses to DUVR between fibroblasts and keratinocytes could also be attributed to wavelength penetration, considering that only UVA reach dermal fibroblasts. It has also been shown that the two distinct stress responses elicited by UVA or UVB may interact leading to a third response, different from either of the two [39]. This may occur in epidermal keratinocytes after DUVR exposure.

Our study also revealed that some responses were similar between both cell types, as shown for some members of the sestrin family and MsrA. We noticed here for the first time that some sestrin genes SESN1-T1 and T2 and SESN3 could be down-regulated by low doses of DUVR in human primary skin cells integrated in a 3D organotypic model. In contrast to our study, SESN1-T2 was described as a rapidly inducible gene by even low doses of hydrogen peroxide in a carcinoma cell line [40]. However, the regulation of sestrin gene expression seems quite complex with a control through p53 depending on the nature of the stress, its intensity, or the sestrin member [19], [40]. This impact may be deleterious since sestrins participate in regeneration of over-oxidized peroxoredoxins, peroxidases responsible for removal of hydrogen peroxide and are essential for reestablishing an antioxidant firewall [20]. This has been illustrated with increased intracellular ROS levels concomitant to down-regulation of SESN1 and SESN3 [41]. This family of genes involved in oxidative stress response obviously needs further experiments to better understand the functional relevance of the modulations. MSRA mRNA levels were also found similarly decreased in both keratinocytes and fibroblasts after DUVR exposure still with a modulation at 24 h, illustrating that a single low dose of DUVR is able to impair the whole cutaneous expression of an important enzyme involved in the maintenance of protein structure and function. Decline of MSRA is strongly associated with aging/photoaging *in vitro* and *in vivo* [42]–[44]. However the regulation of MsrA seems to be complex, depending on the intensity of oxidative stress [22], [43].

In summary, our study illustrates the impact of a low daily UV exposure in oxidative stress response of the skin, even in the absence of any detectable damage at the tissue level and reinforced the notion that the dermal compartment is highly susceptible to such environmental stress.

## Materials and Methods

### Ethics Statement

Normal human breast skin was obtained after written informed consent from healthy subjects following plastic mammary reduction and according to the principles expressed in the Declaration of Helsinki.

### Keratinocyte, fibroblast and reconstructed skin cultures

Epidermal keratinocytes and dermal fibroblasts were isolated from skin samples, cultured and used for reconstruction of normal human reconstructed skin *in vitro*, as described [45].

### Irradiation source and procedure

Daily UV radiation (DUVR) was delivered using a 1000 W Xenon lamp equipped with a dichroic mirror (Oriel, les Ulis, France) and UG5/2 mm thick and WG320 2.6 mm thick filters (Schott, Clichy, France). The spectral irradiance was measured using a spectroradiometer (Macam Photometrics, Livingston, UK) (Figure 1a).

During DUVR exposure, the reconstructed skin medium was replaced by Dulbecco's phosphate-buffered saline (PBS) without calcium and magnesium (Invitrogen, Cergy-Pontoise, France). The control- sham irradiated- samples were not exposed to UV radiation. They were treated identically, being placed in PBS for the same period out of the incubator.

### Histology, immunolabelling and staining

Histology and immunolabelling of vimentin were performed as described [45] using mouse monoclonal antibody against human vimentin (1∶20, Monosan, Unden, the Netherlands) and fluorescein isothiocyanate (FITC)-conjugate rabbit anti-mouse immunoglobulins (1∶80, Dako, Trappes, France) as a second antibody. Nuclear counterstaining using propidium iodide was carried out routinely.

Immunostaining of p53 was performed using mouse monoclonal antibody against human p53 (clone D07, 1∶50, Dako, Trappes, France), goat anti-mouse HRP (1∶100, Dako, Trappes, France) as a second antibody and Dako EnVisions+ System Peroxidase (Dako, Trappes, France) as described [46].

### MMP1 ELISA assay

The amount of secreted interstitial collagenase (MMP1) was assessed in culture medium using the Biotrack MMP-1 human ELISA system (Amersham, England).

### Reactive Oxygen Species (ROS) assay

Reconstructed skins were incubated with 50 µM 2′,7′-dichlorodihydrofluorescein diacetate (DCFH-DA, Invitrogen, Eugen, USA) for 30 min, at 37°C, 5% CO2. After PBS washing, samples were exposed or not to DUVR (7 or 13 J/cm^2^). Immediately after exposure, global fluorescence was quantified in reconstructed skins using a Victor3 fluorimeter (PerkinElmer, Courtaboeuf, France). One hundred fluorescence measures were generated and averaged per sample.

Reconstructed skin samples were then frozen in liquid nitrogen and 5 µm cryostat sections were made and fixed with acetone to allow the visualization of fluorescence generated by ROS in cells of the reconstructed skins.

### Total RNA extraction

Reconstructed skin sample was rinsed in Dulbecco's PBS - (Invitrogen, Cergy-Pontoise, France). The epidermis and dermal equivalent were separated using fine forceps. Disruption of tissue, total RNA extraction and Dnase I treatment were performed using Rneasy midi-kit (Qiagen, Courtaboeuf, France) as described [47]. The absence of contamination between both cell types was checked, using quantitative RT-PCR, by analyzing the levels of mRNA for Col I α1, Col III α1 (fibroblastic genes) in keratinocyte preparations, and for mRNA from laminin β3, a gene specific for keratinocytes in the fibroblast preparation [47]. Histological analysis and immunolabelling for epidermal keratins (using a pan-keratin antibody) were also performed before and after peeling of the epidermis, and revealed that no epidermal contamination could be visualized on the de-epidermized dermal equivalent (data not shown).

### Quantitative reverse transcription PCR

1 µg of total RNA was used for first strand cDNA synthesis using an Advantage RT-for-PCR kit (Clontech, Saint Quentin en Yvelines, France), according to the manufacturer's instructions.

Quantitative PCR was performed using the LightCycler and the LightCycler-FastStart DNA Master Sybr Green kit (Roche Diagnostics, Meylan, France) [48]. Specific parameters of primer sets are given in Table S1. Normalization of data was performed using five housekeeping genes (*b2m*, *G3PDH*, *RPL13A, RPS28, RPS9*) and Genorm application [49], [50].

### Protein extraction

Reconstructed skin sample was rinsed in Dulbecco's PBS - (Invitrogen, Cergy-Pontoise, France). Epidermis and dermis were separated. To obtain whole cell extract, epidermis was lysed in urea buffer (8M). To obtain epidermis cytosolic extracts, Subcellular Proteoextract Kit (Calbiochem, Fontenay-sous-Bois, France) was used. The dermal equivalent was digested with 2 mg/ml Clostridium histolyticum collagenase (Sigma Aldrich, Lyon, France), 45 minutes at 37°C and centrifugated. Whole cell extracts and cytosolic extracts were obtained using Laemmli buffer and Nuclear extract kit respectively (Active Motif, Rixensart, Belgium).

### Western blot analysis

Whole cell or cytosolic extracts were boiled and cleared by centrifugation and the supernatants were separated on Nupage 4-12% bis-tris precast gels (Invitrogen, Cergy-Pontoise, France). Fractionated products were electrotransferred onto Immun-Blot PVDF membranes (Bio-Rad, Marnes-la-Coquette, France). After blocking nonspecific binding sites with 5% nonfat milk, the membranes were incubated using a 1∶1000 dilution of primary antibodies (mouse monoclonal anti-HO1, mouse monoclonal anti-TXNR (OSA11 and SC28321, TEBU Bio, Le Perrey en Yvelines, France), rabbit polyclonal anti-MSRA (07-338, Millipore, Guyancourt, France) or a 1∶2000 dilution for monoclonal anti-GAPDH (ab9484, Abcam, Paris, France). The secondary antibody used were 1∶1000 dilution of polyclonal goat anti-mouse-HRP labeled or 1∶2000 dilution of polyclonal goat anti-rabbit HRP labeled antibody (P0447 and P0448, Dako, Trappes, France). Antigen-antibody complexes were visualized using chemiluminescence detected by a Fluor-S Multi-Imager (Bio-Rad, Marnes-la-Coquette, France).

### Statistical analysis

#### Determination of significant modulations in mRNA amount

For each gene and at each time point, to calculate whether the mean mRNA amount of control samples was statistically different from the mean mRNA amount of irradiated samples, means of the log of mRNA amount were compared using two-tailed Student's *t*-test (p<0.05).

#### Comparison of modulation ratios between fibroblasts and keratinocytes

For each gene and at each time point, 3 modulation ratios were calculated as follows: mRNA amount of irradiated sample to mean mRNA amount of the 3 control samples. The correct homogeneity of variances of the log of modulation ratios was checked. In order to analyze the differences in modulation ratios between fibroblasts and keratinocytes, a two-ways ANOVA test (with Bonferroni's multiple comparison test) was performed on the log of modulation ratios analyzing the significance of group (fibroblasts or keratinocytes) and time (2, 6 or 24 h post exposure). Results were confirmed using Mann-Whitney non parametric test.

## Supporting Information

Table S1 Primers and conditions for quantitative RT-PCR.
(0.09 MB DOC)
