# Supplementary material for: Different Oxidative Stress Response in Keratinocytes and Fibroblasts of Reconstructed Skin Exposed to Non Extreme Daily-Ultraviolet Radiation
Source: PLoS One. 2010 Aug 10;5(8):e12059. doi: 10.1371/journal.pone.0012059 (PMC2919404; doi:10.1371/journal.pone.0012059)
Supplement: Table S1 — Primers and conditions for quantitative RT-PCR. (0.09 MB DOC) [file pone.0012059.s001.doc]

**Table S1. Primers and conditions for quantitative RT-PCR**

|  | genbank accession no | | Gene name | Forward Primer (5'-3') | | Reverse Primer (5'-3') | | Amplicon size (bp) | primer (mM) | Cycles Nb | Annealing temp (°C) |
| --- | --- | --- | --- | --- | --- | --- | --- | --- | --- | --- | --- |
|  | BC001491 | | *HO1* | GAGACGGCTTCAAGCTG | | GTGTGTAGGGGATGACC | | 195 | 0,4 | 40 | 60 |
|  | NM_003330 | | *TXNRD1* | CCTATGTCGCTTTGGAG | | CCCTACGGTTTCTAAGCC | | 323 | 0,4 | 40 | 60 |
|  | NM_000903 | | *NQO1* | CGGCTTTGAAGAAGAAAGG | | CTCGGCAGGATACTGAA | | 131 | 0,4 | 40 | 60 |
|  | NM_001498.2 | | *γ GCS-H* | GCAGAGGAGTACACCC | | CCACTTCCATGTTTTCAAGG | | 109 | 0,4 | 40 | 60 |
|  | NM_002061 | | *γ GCS-L* | TCACCTCCTATTGAAGATGG | | GGTTACTATTTGGTTTTACCTGT | | 174 | 0,4 | 40 | 60 |
|  | NM_000146 | | *FTL* | TCTCGGCCATCTCCTGCTTCTG | | CGCCTTCCAGAGCCACATCATC | | 213 | 0,5 | 40 | 64 |
|  | NM_002032 | | *FTH* | GCCGCCGCCTCTCCTTAGTC | | CAGTTTCTCAGCATGTTCCCTCTCC | | 236 | 0,5 | 40 | 64 |
|  | NM_006164 | | *Nrf2* | CGGTATGCAACAGGACATTG | | GTTGGGGTCTTCTGTGGAGA | | 259 | 0,5 | 40 | 64 |
|  | NM_203500 | | *Keap1* | CACAGCAATGAACACCATCC | | TGTGACCATCATAGCCTCCA | | 227 | 0,5 | 40 | 64 |
|  | NM_001186 | | *Bach1* | TGTGCTTAGAGAAGGATGCTGCTC | | TCTTCGTTTCTTCAGGTTCCATTGC | | 249 | 0,5 | 40 | 64 |
|  | NM_001454 | | *SESN1-T1* | GGCAAACCATTTTGAGGAAA | | TGGTCCCTGTCCTAGTGGTC | | 236 | 0,5 | 40 | 64 |
|  | NM_014454.1 | | *SESN1-T2* | GCTGGGCTGCAAGCAGTG | | CCAAGTTCCTCGTCCTGGT | | 51 | 0,5 | 40 | 64 |
|  | NM_031459 | | *SESN2* | GCACCTACACCCCCTAGTGA | | GTCTTCCACAAAGCACAGCA | | 267 | 0,5 | 40 | 64 |
|  | NM_144665 | | *SESN3* | AGTGCTGCGGAAGGATAAAA | | CCATGCGCAACATGTAAAAC | | 233 | 0,5 | 40 | 64 |
|  | X04076 | | *Catalase* | CATTCGATCTCACCAAGGTTTGGCC | | AGCACGGTAGGGACAGTTCACAGG | | 257 | 0,5 | 40 | 64 |
|  | NM_201397 | | *GSH Px* | GGCTACTCTCTCGTTTCCTTTC | | GTTCTTGGCGTTCTCCTACAG | | 190 | 0,5 | 40 | 64 |
|  | NM_003329 | | *TXN* | CTGCTTTTCAGGAAGCCTTG | | ACCCACCTTTTGTCCCTTCT | | 236 | 0,5 | 40 | 64 |
|  | NM_000454 | | *SOD1* | AGTGCAGGGCATCATCAATTTCGAGCAG | | GATGCAATGGTCTCCTGAGAGTGAGATC | | 298 | 0,5 | 40 | 64 |
|  | NM_000636 | | *SOD2* | GTCACCGAGGAGAAGTACCAGGAG | | CACCAACAGATGCAGCCGTCAG | | 235 | 0,5 | 40 | 64 |
|  | NM_005952 | | *MT1X* | GCTCCTGTGCCTGTGCCG | | AGCAAACGGGTCGGGTTGTAC | | 250 | 0,5 | 40 | 64 |
|  | NM_175617 | | *MT1E* | GCCCGACCTCCGTCTATAA | | AACAAGCAGTCAGGCAGTTG | | 75 | 0,5 | 40 | 64 |
|  | NM_005953 | | *MT2A* | CGCCGCCGGTGACTCCTG | | ACGGTCACGGTCAGGGTTGTAC | | 250 | 0,5 | 40 | 64 |
|  | NM_005950 | | *MT1G* | TCCTGTGCCGCTGGTGTCTC | | ACGGGTCACTCTATTTGTACTTGGG | | 214 | 0,5 | 40 | 64 |
|  | NM_012331 | | *MSRA* | TGGTTTTGCAGGAGGCTATAC | | GTAGATGGCCGAGCGGTACT | | 208 | 0,5 | 40 | 64 |
|  | AB021288 | | *b2m* | TGCTGTCTCCATGTTTGATGTATCT | | TCTCTGCTCCCCACCTCTAAGT | | 86 | 0,4 | 40 | 60 |
|  | M33197 | | *G3PDH* | CAGTCCATGCCATCACTGCCACCCAG | | CAGTGTAGCCCAGGATGCCCTTGAG | | 303 | 0,4 | 34 | 55 |
|  | NM 012423 | | *RPL13A* | CCTGGAGGAGAAGAGGAAAGAGA | | TTGAGGACCTCTGTGTATTTGTCAA | | 126 | 0,4 | 35 | 60 |
|  | BC000354 | | *RPS28* | CGGTACACCTGTCAAACGGTAAC | | TAAGAGGCGTTCATGCATAATCCC | | 507 | 0,4 | 40 | 64 |
|  | NM_001013 | | *RPS9* | GATGAGAAGGACCCACGGCGTCTGTTCG | | GAGACAATCCAGCAGCCCAGGAGGGAC | | 430 | 0,4 | 40 | 64 |
|  | |  | | |  | |  | | | | |
